# A new selenium source from Se-enriched *Cardamine violifolia* improves growth performance, anti-oxidative capacity and meat quality in broilers

**DOI:** 10.3389/fnut.2022.996932

**Published:** 2022-08-29

**Authors:** Xiao Xu, Yu Wei, Yue Zhang, Xiaoqing Jing, Xin Cong, Qingyu Gao, Shuiyuan Cheng, Zhenzhou Zhu, Huiling Zhu, Jiangchao Zhao, Yulan Liu

**Affiliations:** ^1^Hubei Key Laboratory of Animal Nutrition and Feed Science, School of Animal Science and Nutritional Engineering, Wuhan Polytechnic University, Wuhan, China; ^2^Enshi Se-Run Material Engineering Technology Co., Ltd., Enshi, China; ^3^National R&D Center for Se-Rich Agricultural Products Processing, School of Modern Industry for Selenium Science and Engineering, Wuhan Polytechnic University, Wuhan, China; ^4^Department of Animal Science, Division of Agriculture, University of Arkansas, Fayetteville, NC, United States

**Keywords:** *Cardamine violifolia*, broilers, selenium, antioxidant, performance, meat quality

## Abstract

**Background:**

*Cardamine violifolia* (Cv) is a kind of selenium-enriched plant which contains high levels of organic selenium (Se) such as selenocysteine and methylselenocysteine. This study was conducted to investigate the effects of this new source of Se on the growth performance, anti-oxidative capacity and meat quality in broilers compared with other frequently-used Se sources.

**Methods:**

A total of 240 broilers were allotted into 4 treatments: (1) Control group (Se free diets, CON); (2) Sodium selenite group (0.3 mg/kg Se sourced from Na_2_SeO_3_ diets, SeNa); (3) Selenium yeast group (0.3 mg/kg Se sourced from Se-Yeast diets, SeY); (4) Plant Se group (0.3 mg/kg Se sourced from Cv diets, SeCv). The whole study lasted 42 days and was divided into 2 stages (1–21 d as earlier stage and 22–42 d as later stage).

**Results:**

The results showed that the broilers fed SeCv diets had improved average daily gain and the ratio of feed to gain compared to the broilers fed SeNa and SeY diets during the earlier stage. However, there was no significant difference in growth performance of broilers fed these 3 sources of Se diets during the whole period. The broilers fed SeCv diets had improved intestinal mucosal morphology on d 21 and 42. Enhanced liver total anti-oxidative capacity was observed from the broilers fed SeCv diets compared with the other 2 Se sources diets on d 21. Furthermore, lower liver malondialdehyde contents were determined from the broilers fed SeCv and SeY diets compared with SeNa diets. At last, the broilers fed SeCv had increased redness in thigh muscle and decreased cooking loss in both breast and thigh muscle compared with the boilers fed SeNa diets. However, the broilers had similar meat quality between SeCv group and SeY group.

**Conclusion:**

In conclusion, these results demonstrated that SeCv was a well-organic Se source for broilers.

## Introduction

Selenium (Se) is an essential trace element for humans and animals as its crucial functions in antioxidant defense, immune enhancement and so on (1, 2). Se plays its biological function as the core constituent of selenocysteine (SeCys_2_) and selenomethionine (SeMet) which are constituent of antioxidant enzymes such as glutathione peroxidase (GSH-PX) (3, 4). The frequently-used sources of Se for feed additives in poultry diets include sodium selenite (Na_2_SeO_3_) and selenium yeast (SeY). Similar to other trace elements, the bioavailability of Se closely depended on the chemical forms (5). It is generally believed that organic Se sources have lower toxicity, higher bioavailability and better antioxidant properties compared to its inorganic sources (6, 7).

*Cardamine violifolia* (Cv) is a recently discovered Se hyperaccumulating Brassicaceae plant found in Enshi, Hubei, China (8). Cv can efficiently transform inorganic Se into organic Se through its particular metabolic pathways (9). Meanwhile, Cv has a super high Se tolerance and it can accumulate Se above 1,400 mg Se per kg dry weight (10, 11). The main forms of Se existed in Cv are Se-enriched proteins such as SeCys_2_, methylselenocys-teine (MeSeCys) and SeMet (12). The edibility and Se accumulation ability of Cv make it as a potential source of Se supplementation for feed additives. However, there are few studies focused on the effects of Se-enriched Cv (SeCv) on the growth performance and health status in livestock and poultry.

Therefore, this study was aimed to explore the effects of this new Se source—SeCv on the growth performance, anti-oxidative capacity and meat quality in broilers compared with other frequently-used Se sources—SeNa and SeY, respectively.

## Materials and methods

### Animal and experimental design

Animal trial was conducted according to the Animal Scientific Procedures Act 1986 (Home Office Code of Practice. HMSO: London January 1997) and EU regulation (Directive 2010/63/EU). This experimental protocol (No. WPU202111049) used in this study was approved by the Institutional Animal Care and Use Committee of Wuhan Polytechnic University (Wuhan, China). A total of 240 one-day-old, male (initial body weight 45.34 ± 0.67 g) ROSS 308 broiler chicks were obtained from the Xiangyang Charoen Pokphand Co., Ltd., Xiangyang, Hubei, China. All birds were randomly assigned to 1 of 4 dietary treatments with 6 pens per treatment and 10 chicks per pen. The treatments were designed as (1) Control group (Se free diets, CON); (2) Sodium selenite group (0.3 mg/kg Se sourced from Na_2_SeO_3_ diets, SeNa); (3) Selenium yeast group (0.3 mg/kg Se sourced from Se-Yeast diets, SeY); (4) Plant Se group (0.3 mg/kg Se sourced from Cv diets, SeCv). All diets were formulated to meet the nutritional levels for broiler chickens recommended by the National Research Council (1994) (13). The chickens were fed earlier diets from d 1 to 21 and later diets from d 22 to 42. The composition and nutritional level of the experimental diets is shown in Table 1. All chicks were allotted in wire-floored cages (120 ×120 ×60 m^3^) in an environmentally controlled room with continuous light and had *ad libitum* access to feed and water. The ambient temperature was maintained at 36°C at the start of experiment and was decreased as the birds progressed in age. The relative humidity was set at 45–55% and was kept within this range.

**Table 1:** Composition and nutrient contents of basal diets for the broilers (as fed basis).

**Ingredients, %**	**D 1–21 earlier stage**	**D 22–42 later stage**
Corn	55.59	60.80
Soybean meal	36.85	32.50
Soy oil	3.77	3.45
Calcium hydrophosphate	1.65	1.15
Limestone	1.37	1.43
Salt	0.30	0.30
DL-methionine	0.17	0.07
Vitamin and mineral premix^a^	0.30	0.30
**Nutrient contents, %** ^**b**^
Metabolizable energy, kcal/kg	3,000	3,050
Crude protein	21.10	19.54
Ca	1.02	0.92
Non-phytate phosphorus	0.45	0.35
Lysine	1.14	1.03
Methionine	0.50	0.38
Threonine	0.80	0.74

^a^Premix Supplied per kg Diet: Vitamin A, 11,000 IU; Vitamin D, 3,025 IU; Vitamin E, 22 mg; Vitamin K_3_, 2.2 mg; Thiamine, 1.65 mg; Riboflavin, 6.6 mg; Pyridoxine, 3.3 mg; Cobalamin, 17.6 μg; Nicotinic Acid, 22 mg; Pantothenic Acid, 13.2 mg; Folic Acid, 0.33 mg; Biotin, 88 μg; Choline Chloride, 500 mg; Iron, 48 mg; Zinc, 96.6 mg; Manganese, 101.76 mg; Copper, 10 mg; Iodine, 0.96 mg; Cobalt, 0.3 mg. ^b^The Nutrients Contents Were Analyzed Values Except Metabolizable Energy and Non-phytate Phosphorus Which Were Calculated Values.

**Figure 1:**
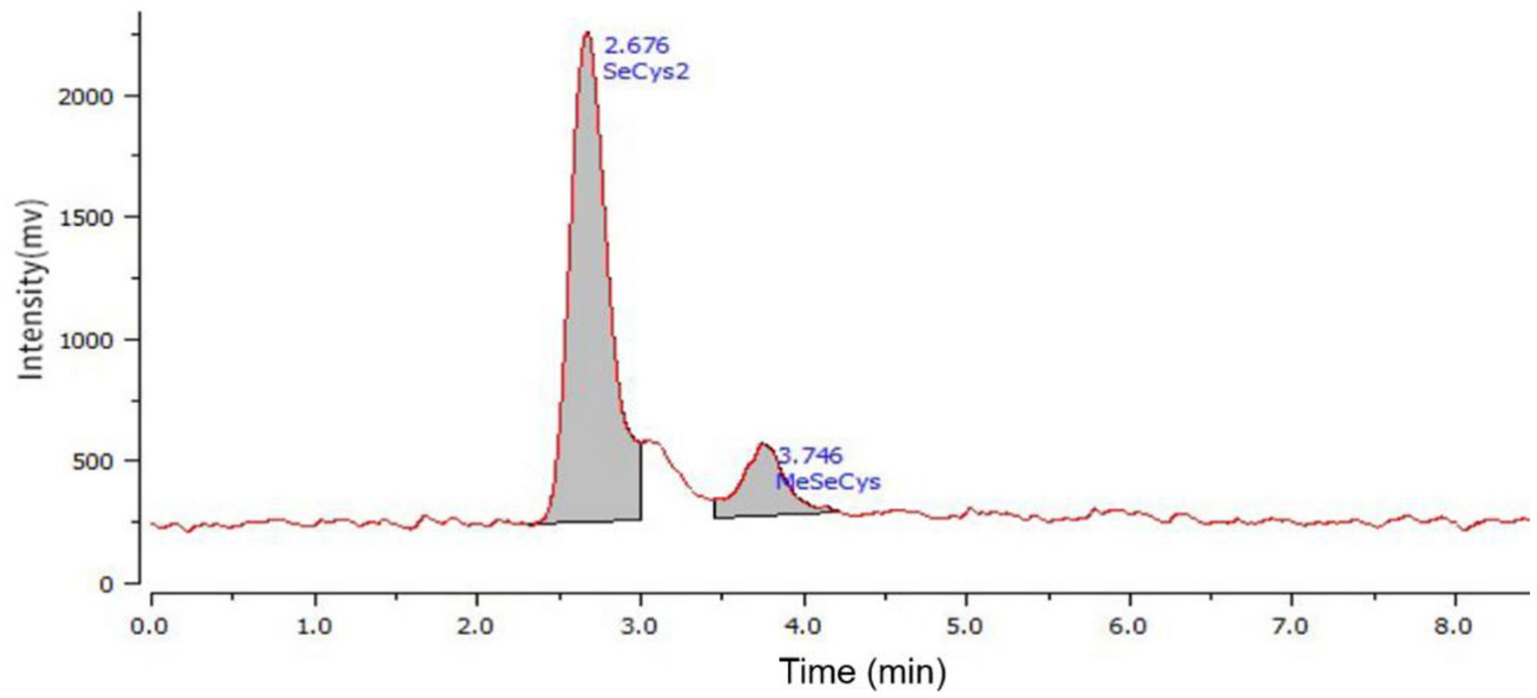
Characterization of selenium existed in Se-enriched *Cardamine violifolia*.

Se-enriched Cv used in this study was provided by Enshi Se-Run Health Tech Development Co., Ltd, Enshi, Hubei, China. This Se-enriched Cv included leaves and stalk with 1,430 mg Se per kg dry weight mainly existed as the forms of SeCys_2_ and MeSeCys determined by the method of high performance liquid chromatography combined with atomic fluorescence (Figure 1). The sodium selenite and selenium yeast samples were commercial products purchased from Angel Yeast Co., Ltd, Yichang, Hubei, China.

### Sample collection

All chicks were weighed individually after their arrival from the hatchery. These birds were also weighed on d 21 and 42. Feed bags were weighed at the same time and these data were used to calculate average daily gain (ADG), average daily feed intake (ADFI) and feed-to-gain ratio (F/G). One bird per cage was randomly selected according to the average weight of the broilers in the cage on d 21 and 42, respectively. The intestine, liver and muscle samples were collected. A 2-cm tissue samples of the duodenum, jejunum and ileum were obtained. The intestinal samples were flushed with 0.9% salt solution, fixed with 10% formaldehyde-phosphate buffer and kept at 4°C for microscopic assessment of intestinal morphology. The liver samples were collected and frozen in liquid nitrogen and transferred to a −80°C freezer for anti-oxidative capacity analysis. The broilers euthanized on d 42 were also used to collect breast and thigh muscle samples. The muscle samples were removed on the left side of the broilers for meat quality determination.

### Intestinal morphology

After a 24 h fixation, the intestinal segments were dehydrated, embedded, and stained with hematoxylin and eosin. Villus height (Vh) and crypt depth (Cd) were measured at 100 × magnification with a microscope (Olympus CX31, Tokyo, Japan) according to our previous study (14). Ten well-oriented and intact villi were selected and determined using a light microscope with a computer-assisted morphometric system (BioScan Opti-metric; BioScan Inc., Edmond, WA, USA). Vh was measured from the tip of the villus to the villus-crypt junction; Cd was defined as the depth of the invagination between adjacent villi.

### Liver anti-oxidative capacity

Total anti-oxidative capacity (T-AOC), activities of glutathione peroxidases (GSH-PX), superoxide dismutases (SOD) and concentrations of malondialdehyde (MDA) in liver were determined by spectrophotometric methods following the instructions of the commercial kits' manufacturer (Nanjing Jiancheng Bioengineering Institute, Nanjing, China) (15).

### Meat quality of the breast and thigh muscles

Meat color, including lightness (L^*^), redness (a^*^), and yellowness (b^*^) values, were measured from 3 locations (middle, medial, and lateral) using a Chromameter (CR-410, Konica Minota, Tokyo, Japan) (16). Drip loss for 24 was measured using the plastic bag method as described previously (17). Briefly, a weight of 30 g of breast or thigh muscle sample was weighed and then put in a sealed plastic bag and kept at 4°C for 24 h. After which the samples were taken out of the bags and dried. The drip loss was the difference of the sample weight to the initial sample weight. Cooking loss was measured by a previous study (18). Briefly, a weight of 30 g sample was weighed and cooked at 80°C for 20 min using a water bath. After cooking, the samples were cooled at room temperature and then weighed. Cooking loss was the difference of the sample weight to the initial weight. Shear force were measured by the methods reported previously (19). Briefly, the cooked samples were taken parallel to muscle fibers to measure maximal shear force (TA500 Lloyd Texture Analyzer fitted with a triangular Warner-Bratzler shear, Lloyd instruments, Bognor Regis, UK).

### Statistical analyses

All data were analyzed as a randomized block design using the general linear model procedures (GLM) of SAS (SAS Inst. Inc., Cary, NC). Statistical significance was declared at *P* < 0.05. If significant effects were found, individual means were compared using Duncan's multiple comparison tests. Data were presented as means and SEMs.

## Results

### Growth performance

As shown in Table 2, the body weight on d 21 of the broilers fed SeCv diets was significantly increased compared with the other 3 groups (*P* < 0.05). During the earlier period, the broilers fed SeCv diets had significantly increased ADG and decreased F/G compared with the other 3 groups (*P* < 0.05). During the later period, the broilers fed the 3 sources of Se diets had no significant difference in growth performance (*P* > 0.05). For the whole feeding period, the broilers fed SeCv diets had significantly increased ADG and decreased F/G compared with the broilers fed CON diets (*P* < 0.05). Similarly, the broilers in SeCv group had a significantly increased body weight on d 42 compared with the broilers in CON group (*P* < 0.05).

**Table 2:** The growth performance of the broilers fed diets containing different sources of selenium.

**Items**	**CON**	**SeNa**	**SeY**	**SeCv**	**SEM**	***P*-value**
D 1 Body weight (g)	45.61	45.12	45.50	45.12	0.67	0.925
D 21 Body weight (g)	645^b^	651^b^	656^b^	726^a^	16	0.027
D 42 Body weight (g)	1982^b^	2037^ab^	2021^ab^	2095^a^	29	0.043
**D 1–21**
Average daily gain (g/d)	28.54^b^	28.87^b^	29.07^b^	32.40^a^	0.83	0.025
Average daily feed intake (g/d)	44.20	42.72	42.33	42.53	1.13	0.735
Feed to gain ratio	1.55^a^	1.48^a^	1.46^a^	1.32^b^	0.05	0.012
**D 22–42**
Average daily gain (g/d)	63.66	65.98	64.99	65.21	1.22	0.187
Average daily feed intake (g/d)	117.4	114.1	114.4	114.8	1.16	0.325
Feed to gain ratio	1.85^a^	1.73^b^	1.76^ab^	1.76^ab^	0.03	0.038
**D 1–42**
Average daily gain (g/d)	46.10^b^	47.43^ab^	47.03^ab^	48.81^a^	0.68	0.046
Average daily feed intake (g/d)	80.81	78.39	78.34	78.67	0.99	0.568
Feed to gain ratio	1.75^a^	1.65^b^	1.67^b^	1.61^b^	0.02	0.002

*N* = 6 (10 birds per cage). The same letter on the shoulder of the mean in the same line indicates that the difference is insignificant, and the absence of the same letter means that the difference is significant. SEM, standard error of mean.

### Intestinal morphology

**Figure 2:**
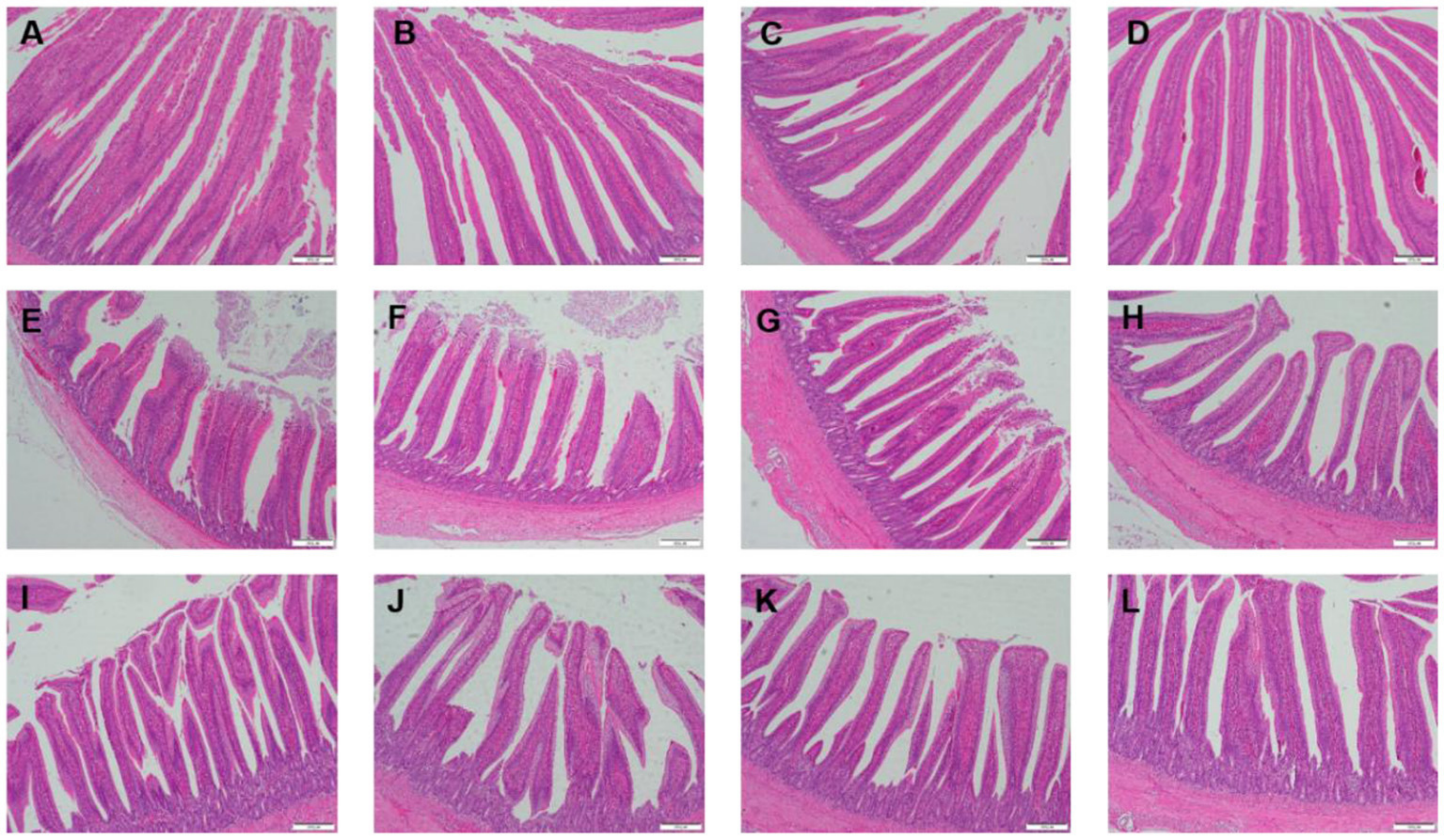
Intestinal mucosal histological appearance (hematoxylin and eosin) of the broilers fed diets containing different sources of selenium on D 21. Original magnification 100 ×. Scale bars = 200 μm. **(A–D)** Duodenum histological appearance of the broilers fed CON, SeNa, SeY, and SeCv diets, respectively. **(E–H)** Jejunum histological appearance of the broilers fed CON, SeNa, SeY, and SeCv diets, respectively. **(I–L)** Ileum histological appearance of the broilers fed CON, SeNa, SeY, and SeCv diets, respectively.

**Figure 3:**
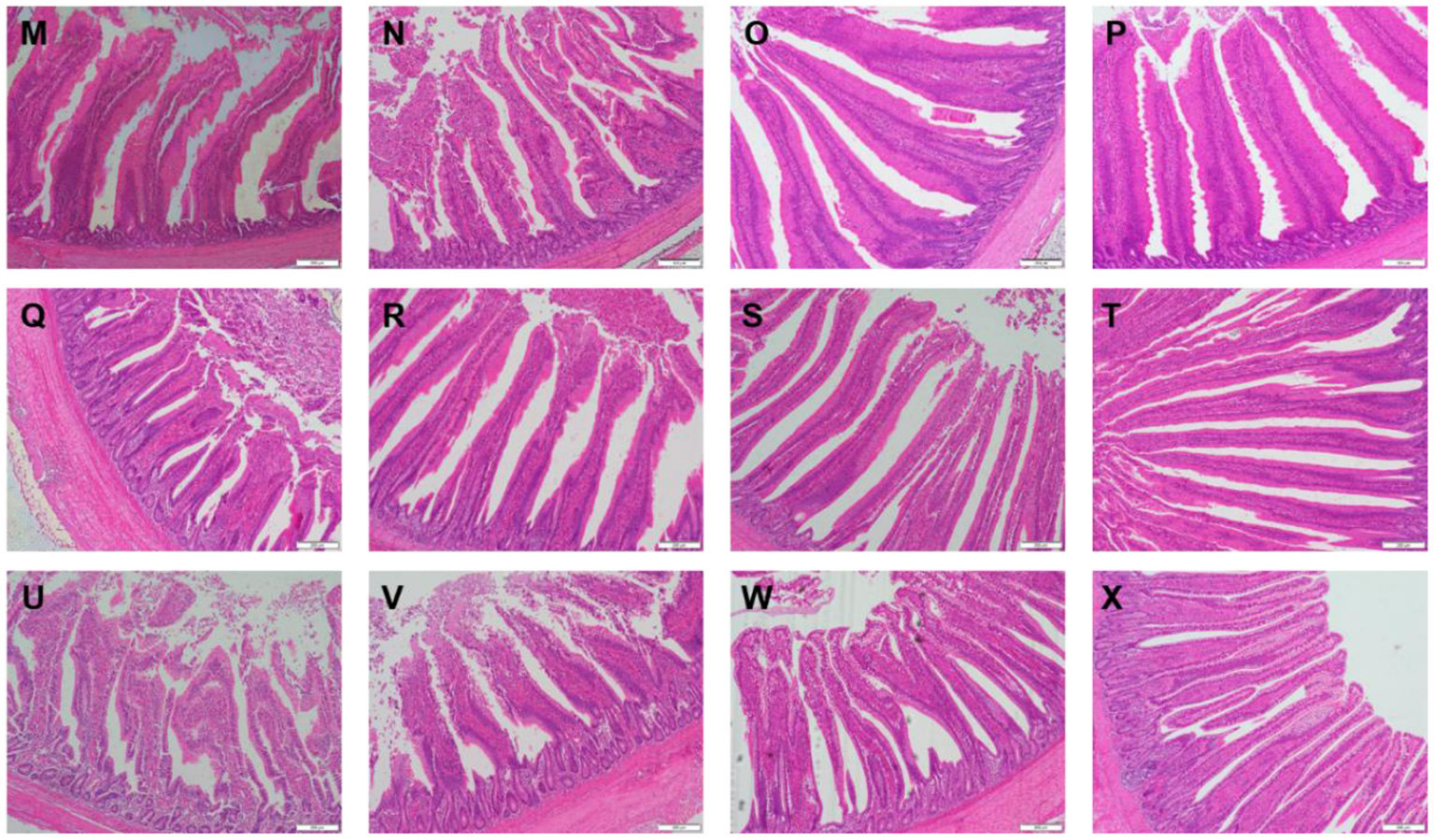
Intestinal mucosal histological appearance (hematoxylin and eosin) of the broilers fed diets containing different sources of selenium on D 42. Original magnification 100 ×. Scale bars = 200 μm. **(M–P)** Duodenum histological appearance of the broilers fed CON, SeNa, SeY, and SeCv diets, respectively. **(Q–T)** Jejunum histological appearance of the broilers fed CON, SeNa, SeY, and SeCv diets, respectively. **(U–X)** Ileum histological appearance of the broilers fed CON, SeNa, SeY, and SeCv diets, respectively.

Table 3 showed the intestinal morphology of the broilers fed diets containing different sources of Se. On d 21, Vh and Vh/Cd of duodenum and ileum in the broilers fed SeCv diets were significantly increased compared with the broilers fed the other 3 diets (*P* < 0.05). Vh and Vh/Cd of jejunum in the broilers fed SeCv diets were significantly increased compared with the broilers fed CON and SeY diets (*P* < 0.05). On d 42, the broilers fed SeCv diets had significantly increased Vh and Vh/Cd of duodenum compared with the other 3 groups (*P* < 0.05). Jejunal Vh and Vh/Cd in the broilers fed SeCv diets were significantly increased compared with the broilers fed CON and SeNa diets (*P* < 0.05). Ileal Vh in the broilers fed SeCv diets were significantly increased compared with the other 3 groups (*P* < 0.05). And ileal Vh/Cd in the broilers fed SeCv diets were significantly increased compared with the CON group (*P* < 0.05). Similar to the above results, the histological appearance showed that dietary SeCv alleviated intestinal mucosal injury of the broilers compared with the other 3 groups on d 21 and 42 (Figures 2, 3).

**Table 3:** The intestinal morphology of the broilers fed diets containing different sources of selenium.

**Items**	**CON**	**SeNa**	**SeY**	**SeCv**	**SEM**	***P*-value**
**D 21**
**Duodenum**
Villus height (μm)	889^c^	1003^b^	1035^b^	1173^a^	26	<0.001
Crypt depth (μm)	223	244	255	263	13	0.158
Villus height/crypt depth	3.99^b^	4.11^b^	4.06^b^	4.46^a^	0.13	0.018
**Jejunum**
Villus height (μm)	622^c^	777^ab^	757^b^	827^a^	24	0.008
Crypt depth (μm)	168	175	181	185	12	0.420
Villus height/crypt depth	3.70^c^	4.44^ab^	4.18^b^	4.47^a^	0.14	0.042
**Ileum**
Villus height (μm)	615^c^	617^c^	693^b^	767^a^	28	<0.001
Crypt depth (μm)	180	174	190	194	12	0.625
Villus height/crypt depth	3.42^b^	3.55^b^	3.65^b^	3.95^a^	0.12	0.003
**D 42**
**Duodenum**
Villus height (μm)	817^c^	935^b^	1002^b^	1143^a^	35	<0.001
Crypt depth (μm)	274	274	248	251	16	0.418
Villus height/crypt depth	2.98^d^	3.41^c^	4.04^b^	4.55^a^	0.16	<0.001
**Jejunum**
Villus height (μm)	756^c^	881^b^	963^a^	989^a^	31	0.020
Crypt depth (μm)	252	263	268	267	14	0.622
Villus height/crypt depth	3.00^c^	3.35^b^	3.59^ab^	3.70^a^	0.15	0.017
**Ileum**
Villus height (μm)	625^c^	684^bc^	701^b^	773^a^	28	<0.001
Crypt depth (μm)	194	203	212	219	14	0.254
Villus height/crypt depth	3.22^b^	3.37^ab^	3.31^ab^	3.53^a^	0.14	0.038

*N* = 6 (1 bird per cage). The same letter on the shoulder of the mean in the same line indicates that the difference is insignificant, and the absence of the same letter means that the difference is significant. SEM, standard error of mean.

### Liver anti-oxidative capacity

On d 21, the liver T-AOC of broilers fed SeCv diets was significantly increased compared with the other 3 groups (*P* < 0.05, Table 4). The activities of GSH-PX and SOD had no significant difference among the broilers fed different sources of Se (*P* > 0.05). However, the broilers fed the 3 sources of Se diets all had significantly increased activities of GSH-PX and SOD compared with the broilers fed CON diets (*P* < 0.05). Similarly, the broilers fed the 3 different sources of Se all had significantly reduced MDA content (*P* < 0.05). On d 42, the liver T-AOC of broilers fed SeCv diets was significantly increased compared with CON and SeNa group (*P* < 0.05). The activities of GSH-PX and SOD results were similar to the results on d 21. The broilers fed the 3 sources of Se diets all had significantly increased activities of GSH-PX and SOD compared with the broilers fed CON diets (*P* < 0.05). Liver MDA content in the broilers fed SeCv diets were significantly decreased compared with the broilers fed CON and SeNa diets (*P* < 0.05).

**Table 4:** The liver anti-oxidative capacity of the broilers fed diets containing different sources of selenium.

**Items**	**CON**	**SeNa**	**SeY**	**SeCv**	**SEM**	***P*-value**
**D 21**
T-AOC (mM/g)	0.165^b^	0.173^b^	0.182^b^	0.247^a^	0.011	<0.001
GSH-PX (U/mg)	20.3^b^	55.7^a^	56.8^a^	56.6^a^	3.7	<0.001
SOD (U/mg)	129^b^	148^a^	151^a^	159^a^	3	<0.001
MDA (nmol/mg)	2.13^a^	1.62^b^	1.48^b^	1.60^b^	0.08	0.012
**D 42**
T-AOC (mM/g)	0.479^b^	0.472^b^	0.513^a^	0.524^a^	0.013	<0.001
GSH-PX (U/mg)	20.8^b^	48.6^a^	49.9^a^	52.9^a^	2.9	<0.001
SOD (U/mg)	145^b^	151^a^	145^b^	156^a^	2	0.044
MDA (nmol/mg)	1.72^b^	2.20^a^	1.30^c^	1.17^c^	0.09	<0.001

*N* = 6 (1 bird per cage). The same letter on the shoulder of the mean in the same line indicates that the difference is insignificant, and the absence of the same letter means that the difference is significant. GSH-PX, glutathione peroxidases; MDA, malondial-dehyde; SEM, standard error of mean; SOD, superoxide dismutase; T-AOC, total antioxidant capacity.

### Meat quality of breast and thigh muscles

The redness of breast muscle in the broilers fed SeCv diets was significantly higher compared with the broilers fed CON diets (*P* < 0.05, Table 5). The broilers fed the 3 sources of Se diets had no significantly difference in redness, drip loss and shear force in breast muscle (*P* < 0.05). Compared with the broilers fed CON diets, the broilers fed SeCv diets had decreased drip loss, cooking loss as well as shear force (*P* < 0.05). As for thigh muscle, dietary SeCv significantly improved redness compared with the other 3 groups (*P* < 0.05). The broilers fed SeCv diets had significantly reduced drip loss in thigh muscle compared with broiler fed CON diets (*P* < 0.05). Moreover, the broilers fed SeCv diets had significantly reduced shear force in thigh muscle compared with broiler fed CON and SeNa diets (*P* < 0.05).

**Table 5:** The meat quality of the breast and thigh muscle in the broilers fed diets containing different sources of selenium.

**Items**	**CON**	**SeNa**	**SeY**	**SeCv**	**SEM**	***P*-value**
**Breast muscle**
**Color**
L*	59.22	61.00	62.78	62.61	2.74	0.623
a*	11.22^b^	12.25^ab^	12.32^ab^	14.65^a^	0.85	0.012
b*	4.53	4.44	5.14	5.32	0.52	0.674
Drip loss (%)	1.72^a^	1.38^ab^	1.04^ab^	0.80^b^	0.33	<0.001
Cooking loss (%)	33.46^a^	31.58^ab^	29.65^bc^	27.32^c^	1.22	0.018
Shear force (N)	32.35^a^	28.71^b^	27.89^b^	26.94^b^	1.31	0.005
**Thigh muscle**
**Color**
L*	64.58	63.50	65.85	66.24	2.44	0.910
a*	14.58^b^	14.26^b^	15.66^b^	18.32^a^	0.85	<0.001
b*	5.76	6.25	6.47	6.25	0.60	0.509
Drip loss (%)	1.16^a^	1.11^ab^	0.87^ab^	0.58^b^	0.24	0.021
Cooking loss (%)	31.69^a^	31.89^a^	30.96^ab^	28.52^b^	1.12	0.012
Shear force (N)	15.86	16.36	15.02	15.77	0.77	0.413

*N* = 6 (1 bird per cage). The same letter on the shoulder of the mean in the same line indicates that the difference is insignificant, and the absence of the same letter means that the difference is significant. SEM, standard error of mean.

## Discussion

The hypothesis that the new source of Se from SeCv has equal or exceeded effects compared with Se sources from SeNa and SeY on growth performance, anti-oxidative capacity and meat quality of broilers was partly supported by the results that the broilers fed SeCv diets had increased liver T-AOC and reduced MDA content, improved intestinal morphology and reduced cooking loss of breast and thigh muscle than broilers fed SeNa diets. Furthermore, majority of the results in this study showed the similar effects on broilers fed the diets between SeCv and SeY. Therefore, these findings provided scientific experimental bases for improving growth performance and meat quality of broilers with a new choice of Se sources.

Se is one of the substantial trace elements for animals. It promotes growth and plays important roles in maintaining normal development and production of animals (20, 21). Generally, the Se concentration in raw feed material is too low to satisfy the needs of animals for development and health. So, Se as feed additives should be supplemented by external sources (22). Se is not only a functional nutrient but also a high toxic mineral element. In China, the limit of Se in mixed feed for poultry is 0.5 mg/kg (23). Se can be simply divided into organic and inorganic sources. Inorganic Se is mainly used in the form of SeNa, which has an economic advantage and is most widely used in animal diets. However, its strong toxicity, low bioavailability, and oxidation potential have adverse effects on animals and the environment (24). Several studies reported that organic Se existed in the form of SeY had good absorption and utilization rates (4, 25). In this current study, the broilers fed SeCv diets showed an improved performance in the earlier stage and similar performance in the whole period compared with the other 2 Se sources diets. Cv is a hyperaccumulating plant newly found in China. It can transfer inorganic Se from the soil into organic Se mainly existed by the form of SeCys_2_ and MeSeCys. The similar growth performance among these groups demonstrated that SeCv was a well-replacement of other frequently-used Se sources such as SeNa and SeY for broilers.

Intestinal healthy status can be reflected by a series of indicators such as Vh and Cd (26). Vh and Cd are the most intuitive indicators reflecting the morphological and structural integrity of intestinal mucosa (27). Villi are the main components responsible for nutrient absorption in the small intestine, and increased Vh and Vh/Cd can lead to greater absorption of nutrients and improve growth performance (28). In this study, the intestinal Vh and Vh/Cd of broilers fed different sources of Se was improved compared with the broilers fed Se free diets. And the SeCv diets showed the best effects on the intestinal morphology of the broilers among these groups. In agreement with our study, many previous research reported that Se supplementation could improve intestinal Vh and Vh/Cd of animals (29, 30). Se as the key component of several anti-oxidative enzymes, plays a significant role in gut epithelial cell protection from pro-apoptotic oxidant stress, which in turn enhances their growth and development (31). The superiority of SeCv to SeNa may be caused by the capacity of SeNa to bind to the lines of epithelial tissues in the intestinal lumen, thereby being inaccessible for assimilation and transfer to tissues (32).

The antioxidant system of broilers is regulated through many important anti-oxidative enzymes, including GSH-PX, SOD and so on (33). T-AOC reflects the body or organ cumulative effect of all antioxidants (34). MDA is an important marker for reflecting the degree of lipid peroxidation and the extent of cellular damage (35). As the rapid progress of poultry breeding, although the broilers show improved performance, they are easily attacked by external stressors such as high density and anti-nutritional factors which leading to excessive production of reactive oxygen species (36). The antioxidant effect of Se is generally achieved by GSH-PX, because Se is a component of its active center element (37). This study showed the similarly positive effects of SeCv and SeY on liver anti-oxidative capacity of broilers which is superior to the broilers fed SeNa and CON diets. Some research demonstrated that organic Se was more effective than inorganic Se in improving anti-oxidative capacity (38). The reason of similar results of SeCv and SeY may be that the 2 sources of Se were both mainly existed in the form of Selenoprotein (4, 39).

Meat color, drip loss, cooking loss and shear force are all important indicators for reflecting the meat quality (40). There were studies demonstrated that Se could increase the capacity of oxidation resistance, prevent the myoglobin or oxymyoglobin being oxidized to metmyoglobin, deepen the muscle chroma, increase meat redness, and improve meat quality (41, 42). Drip loss and cooking loss reflect the ability of muscle proteins to attract water and hold it within the cells (43). Shear force reflects the tenderness which is associate with the mouthfeel of the meat (44). When the animals were under acute or chronic stress, the above parameters of meat quality usually increased (45). In this study, the broilers fed SeCv diets had higher thigh muscle redness compared with other Se sources diets and similar scores of drip loss, cooking loss and shear force compared with SeY diets. These results were in accordance with the growth performance and anti-oxidative capacity which illustrated that SeCv was a well-additive to improve meat quality of breast and thigh muscle of broilers and had a similar effect to SeY.

## Conclusion

In conclusion, the broilers fed SeCv diets had a better effect compared with SeNa and a similar effect compared with SeY on growth performance, anti-oxidative capacity and meat quality. These results recommended that SeCv was a well Se source for broilers diets.

## Data availability statement

The original contributions presented in the study are included in the article/supplementary files, further inquiries can be directed to the corresponding author/s.

## Ethics statement

The animal study was reviewed and approved by the Institutional Animal Care and Use Committee of Wuhan Polytechnic University (Wuhan, China). The chicks used in the study were obtained from the Xiangyang Charoen Pokphand Co., Ltd. (Xiangyang, China). Written informed consent was obtained from the owners for the participation of their animals in this study.

## Author contributions

XX and YW wrote the manuscript. YZ, XJ, XC, QG, SC, ZZ, HZ, JZ, and YL read and approved the final version. All authors were involved in study design and implementation, data acquisition, analysis, and interpretation.

## Funding

This research was financially supported by the Technology Reserve Project from School of Modern Industry for Selenium Science and Engineering, Wuhan Polytechnic University (Sel-202108), the Scientific Research Project of Wuhan Polytechnic University (2022J05), and the Key Research and Development Program of Hubei Province (2020BBA043).

## Conflict of interest

Authors YZ, XC, and QG were employed by the company Enshi Se-Run Material Engineering Technology Co., Ltd. Enshi, China. The remaining authors declare that the research was conducted in the absence of any commercial or financial relationships that could be construed as a potential conflict of interest.

## Publisher's note

All claims expressed in this article are solely those of the authors and do not necessarily represent those of their affiliated organizations, or those of the publisher, the editors and the reviewers. Any product that may be evaluated in this article, or claim that may be made by its manufacturer, is not guaranteed or endorsed by the publisher.

## Abbreviations

ADFI: average daily feed intake
ADG: average daily gain
BW: body weight
Cd: crypt depth
Cv: *Cardamine violifolia*
F/G: feed-to-gain ratio
GSH-PX: glutathione peroxidases
MDA: malondialdehyde
ROS: reactive oxygen species
SOD: superoxide dismutases
T-AOC: total anti-oxidative capacity
Vh: villus height.
